# Cyclodialysis Clefts following Microinvasive Glaucoma Surgery with Consecutive Intraocular Pressure Spikes

**DOI:** 10.1155/2022/7595507

**Published:** 2022-10-19

**Authors:** Ahmed Alshaikhsalama, Niraj Nathan

**Affiliations:** Department of Ophthalmology, UT Southwestern Medical Center, Dallas, TX, USA

## Abstract

The purpose of this study is to report a case of cyclodialysis clefts associated with microinvasive glaucoma surgery (MIGS) having two distinct consecutive IOP spikes during cleft closure. A 65-year-old female with a history of primary open angle glaucoma was evaluated for right eye blurry vision since cataract surgery 11 months prior. She reported a MIGS procedure that resulted in a cyclodialysis cleft, with resultant hypotony without resolution. On gonioscopy, two clock hours of widened angle were noted nasally, with small clefts (0.5 clock hour) inferonasally and superonasally. Conservative therapy with cycloplegia was unsuccessful. Argon laser photocoagulation was performed for cleft closure. Initially, while there was visible improvement in the cleft size, it did not close completely, and IOP remained low. Additional laser was performed, one week following, she presented with an acute IOP increase to 55 mmHg. On gonioscopy, it was noted that despite the IOP spike indicating inferonasal closure, the superonasal cleft remained small but open. She was started on IOP-lowering therapy. Her IOP normalized shortly thereafter. Two weeks later, she experienced another acute IOP spike to 54 mmHg. On gonioscopy, the residual cleft had closed. Again, her IOP normalized shortly after and has remained normal since. While IOP spikes after cyclodialysis cleft closure have been reported many times, two consecutive IOP spikes of similar magnitude during sequential closure of two concurrent cyclodialysis clefts have not been reported in the literature. This case raises interesting questions about the physiology underlying an acute increase in IOP following cleft closure.

## 1. Introduction

Cyclodialysis is rare and usually presents after blunt trauma or as a complication from surgery and can cause a decrease in intraocular pressure < 5 mmHg [1]. It can be diagnosed via gonioscopy (gold standard) as well as anterior segment OCT and ultrasound biomicroscopy (UBM) [2]. In many cases, a cyclodialysis cleft (CDC) can resolve spontaneously or with conservative therapy; however, laser and surgical intervention can be performed for cases that fail conservative management [3]. Once the cyclodialysis cleft resolves, a spike in IOP (often as high as 70 mmHg) often occurs [4]. The reasons for this increase in IOP following cleft closure are unclear, but multiple mechanisms have been hypothesized. It could be due to a compensatory upregulation of ciliary body function to counteract the increased uveoscleral outflow caused by the cleft; it could also be due to disrupted drainage function through the trabecular meshwork and Schlemm's canal due to collapse or fibrosis while outflow is preferentially through the cleft [4]. With both mechanisms, the thought is that with sudden closure of the cleft, these compensatory changes take time to normalize, with the result being elevated intraocular pressure until they do. In this case report, we present a patient with two discrete cyclodialysis clefts in one eye that closed at separate times despite simultaneous treatment, resulting in two separate IOP spikes of equal magnitude. This course of two discrete consecutive IOP spikes of similar magnitude has not been previously reported and raises interesting questions about the mechanism of IOP increase after cyclodialysis cleft closure.

## 2. Material Summary

This case report examines a patient with a cyclodialysis cleft following microinvasive glaucoma surgery that caused a repeated IOP spike. The cyclodialysis cleft was monitored with gonioscopy. Following partial cleft closure, the IOP peaked to 55 mmHg. After a 2-week period of controlled IOP and therapy, an equal IOP elevation was observed, which coincided with complete cleft closure on gonioscopy. Thus, it is hypothesized that the increase in IOP could be a reaction to cleft closure beyond compensatory mechanisms.

## 3. Case Report/Case Presentation

A 65-year-old female with a history of primary open angle glaucoma was evaluated for right eye blurry vision since cataract surgery 11 months prior. She reported a microinvasive glaucoma procedure (MIGS) was attempted at the time and resulted in a cyclodialysis cleft, with resultant hypotony that had not resolved since. Prior to her procedure, she had a history of primary open angle glaucoma, mild in the right eye, and moderate in the left eye with maximum IOP in the 20s, per her recollection. She was on latanoprost at bedtime and dorzolamide-timolol twice daily in both eyes. Since the procedure in the right eye, her IOP remained in the 4 to 6 mm Hg range, even after the cessation of her IOP lowering drops in that eye. The fellow eye underwent cataract surgery without a MIGS procedure in the interval prior to presentation, complicated by elevated IOP following, so brimonidine twice daily was added to the left eye.

**Figure 1:**
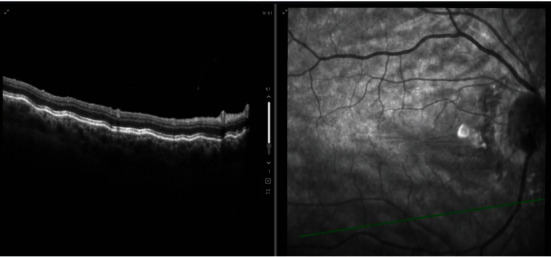
Spectralis OCT of the right macula showing choroidal folds.

On gonioscopy, two clock hours of widened angle were noted nasally, with small cyclodialysis clefts (approximately 0.5 clock hour) on each side of the widened area, inferonasally and superonasally. On exam, she was noted to have uncorrected visual acuity of 20/50-2 with a manifest refraction of plano +2.25 × 090, suggesting her toric was IOL 90 degrees malrotated, which was eventually confirmed by keratometry. She had an IOP of 4 mmHg in the affected eye and 19 mmHg in the fellow eye. She was noted to have 0.5+ anterior chamber cell. Mild choroidal folds were noted on exam and OCT (shown in Figure 1). Best corrected visual acuity was 20/25 + 2, suggesting that most of her blur was related to her malrotated toric lens, but she noted subjectively that even with the correction it seemed blurry still. Still, it was felt most prudent that the hypotony should be addressed prior to attempting intraocular surgery to rotate her toric IOL.

Given her anterior chamber inflammation, a trial of topical steroids and cycloplegia was performed, first with lotemax four times daily then with prednisolone, given her history of significant steroid response, along with atropine twice daily. After 3 months of attempted conservative therapy, her IOP remained low at 4 mmHg with continued choroidal folds on OCT. The decision was made to attempt argon laser photocoagulation to close the cleft with continuation of cycloplegia. Laser therapy (800-830 milliwatts, 500 millisecond duration, 200-micron spot size, 38 total spots) was performed with laser applied to visible cleft openings and surrounding sclera and iris to close the clefts. Two weeks post laser therapy, on gonioscopy, the clefts appeared smaller but not closed, and IOP remained low at 4 mmHg, so a second session of laser was performed (900 milliwatts, 500 millisecond duration, 200-micron spot size, and 50 total spots).

One week later, the patient began experiencing pain above the right eyebrow; her IOP was 26 mmHg, which was notable since this was the first time her IOP was above 6 mmHg in over a year. She was started on dorzolamide-timolol twice daily. The next day, the patient presented to the ER in the night with increased ocular pain, vomiting, and cloudy vision with an IOP of 55 mmHg. She was advised to add latanoprost at bedtime, brimonidine three times daily, and oral Diamox 500 mg twice daily. She was seen the following day with IOP down to 14 mmHg. On gonioscopy, her clefts were mostly closed but one small portion of the superonasal cleft was still visibly not closed. Diamox was held, but topical therapy with latanoprost, brimonidine, and dorzolamide-timolol was continued to be safe. On follow up a week later, her IOP was 7 mmHg. Only brimonidine was stopped, to reduce the risk of IOP increase, and she continued on latanoprost at bedtime and dorzolamide-timolol twice daily.

Two weeks later, the patient abruptly developed right eye pain, cloudy vision, and nausea with an IOP of 54 mmHg. An anterior chamber paracentesis was performed that reduced the pressure down to 14 mmHg; topical brimonidine and oral Diamox were added again. On gonioscopy, the superonasal cleft was now noted to be completely closed. At subsequent follow up visits, the patient's IOP remained stable around 20 mmHg on latanoprost, dorzolamide-timolol, and brimonidine. The choroidal folds on exam resolved and did not recur. Numerical hypotony did not recur as well. She subsequently underwent rotation of her toric IOL and then YAG capsulotomy, with subsequent improvent in her incorrect visual acuity from 20/60-2 to 20/20-1. She was eventually able to be tapered off brimonidine, with IOP 14-15 mmHg in each eye.

## 4. Discussion

CDC is a rare consequence of trauma and/or surgery. It involves a separation between the ciliary muscle and the scleral spur enabling aqueous humor to flow into the suprachoroidal space resulting in ocular hypotension. Persistent hypotony can lead to permanent vision loss, and so it is important to be corrected [3, 5]. Once the CDC closes, a spike in IOP is frequently observed [4]. Although transient, an increase in IOP can cause eye pain, retinal vascular occlusion, ischemic ocular neuropathy, and glaucoma progression [6].

Our case presents as an eye with two cyclodialysis clefts as a complication of microinvasive glaucoma surgery. In this case, the clefts closed sequentially, leading to two discrete acute IOP spikes of similar magnitude (55/54 mmHg).

With the prevailing theories of IOP spike after cyclodialysis cleft closure being due to compensation for hypotony/over filtration by the cleft either by increased aqueous production or decreased trabecular outflow, it is reasonable to hypothesize that if these mechanisms had at least partially normalized to the point of normotension after the first spike, albeit on topical medications; then, the second spike should likely have been of less magnitude than the first, given some renormalization of aqueous production and trabecular outflow, even if partial, if this is indeed the mechanism underlying cleft closure-related IOP spikes.

Two points are important to note to support this hypothesis. First, in this instance, the second IOP spike is very unlikely to be related directly to tapering of medications after the first spike, as the medications were tapered very conservatively (one agent at a time over multiple visits), and the patient presented with an acute (within hours) symptomatic IOP rise two weeks after the tapering of a single drop, while still on three topical IOP-lowering medications. Further, the acute rise coincided with the closure of the residual second cleft on gonioscopy.

Second, it is possible the use topical antihypertensives at the time of the second spike could have masked some ongoing increased aqueous production and/or impaired trabecular function, despite the patient being normotensive; that is, that the traditional aqueous production and trabecular outflow had not fully normalized due to the residual cleft or due to timing. That being said, the prevailing theory is that the eye readjusts these compensatory mechanisms after cleft closure, which accounts for resolution of the IOP spike, typically allowing for cessation of the increased IOP lowering therapy.

If some trabecular outflow had been restored and/or aqueous production reduced more to typical physiologic levels, it would seem to suggest that one would have expected less of an IOP spike the second time, when the eye was no longer hypotonous and had returned at least some trabecular outflow restored after resolution of the initial IOP spike even allowing tapering of medications over multiple visits. It is impossible to say if the patient's preexisiting glaucoma and presumably impaired outflow facility at baseline could have played a role in the phenomenon; it remains a worthy consideration.

In the authors' opinions, this patient's course perhaps suggests that IOP spike may be a direct reaction to cleft closure itself and not just related to compensatory changes in aqueous production and trabecular outflow while the cleft is open. We feel this warrants further investigation as to what physiologic processes mediate this IOP response and resolution.

## 5. Conclusions

IOP spikes are a frequent consequence of CDC and can be dangerous. Two consecutive IOP spikes of similar magnitude during sequential cleft closure have not been reported in the literature and could be important for understanding the physiology underlying an acute increase in IOP. Understanding these mechanisms and the associated factors may allow better prevention and treatment. Further studies are required to investigate the mechanisms underlying an IOP spike in patients after CDC closure.

## Data Availability

The data that support the findings of this study are not publicly available due to being extracted from the patient's chart from our institutional electronic health record system. However, any requests can be directed to the corresponding author NN.

## References

[1] Mushtaq B., Chiang M. Y., Kumar V., Ramanathan U. S., Shah P. (2005). Phacoemulsification, persistent hypotony, and cyclodialysis clefts. *Journal of Cataract and Refractive Surgery*.

[2] Ioannidis A. S., Barton K. (2010). Cyclodialysis cleft: causes and repair. *Current Opinion in Ophthalmology*.

[3] Ioannidis A. S., Bunce C., Barton K. (2014). The evaluation and surgical management of cyclodialysis clefts that have failed to respond to conservative management. *The British Journal of Ophthalmology*.

[4] Provencher L. M., Shah M. M. (2020). Cyclodialysis cleft repair with goniotomy for the control of post-operative ocular hypertension. *American Journal of Ophthalmology Case Reports*.

[5] Thomas M., Vajaranant T. S., Aref A. A. (2015). Hypotony maculopathy: clinical presentation and therapeutic methods. *Ophthalmology and Therapy*.

[6] Fang E. N., Kass M. A. (1994). Increased intraocular pressure after cataract surgery. *Seminars in Ophthalmology*.

